# Supplementary material for: Spectrally specific temporal analyses of spike-train responses to complex sounds: A unifying framework
Source: PLoS Comput Biol. 2021 Feb 22;17(2):e1008155. doi: 10.1371/journal.pcbi.1008155 (PMC7932515; doi:10.1371/journal.pcbi.1008155)
Supplement: S1 Text — (PDF) [file pcbi.1008155.s001.pdf]

S1 Text. Classic metrics for quantifying temporal coding in the auditory system

Various approaches and metrics have been developed to quantify auditory temporal coding in neurophysiological responses. In this section, we motivate the need for a unified framework for auditory temporal coding by briefly reviewing these classic metrics and discussing their benefits and limitations.

Period-histogram based metrics

The ability of AN fibers to follow the temporal structure of an acoustic stimulus has been known for a long time (Galambos and Davis, 1943). Using tones as stimuli, Kiang and colleagues showed that AN fibers prefer to discharge spikes around a particular phase of the stimulus cycle (Kiang et al., 1965). Their analysis was qualitative and involved the period histogram, which is constructed as the histogram of spike times modulo the period of one stimulus cycle (e.g., Fig 1D). Rose and colleagues used the period histogram to quantify the preference of neurons to fire during one half-cycle of a periodic stimulus (Rose et al., 1967). They introduced a metric, called the coefficient of synchronization, which is defined as the ratio of the spike count in the most effective half-cycle to the spike count during the whole stimulus cycle. The coefficient of synchronization ranges from 0.5 (for a flat period histogram) to 1.0 (for all spikes within one half-cycle). The coefficient of synchronization does not truly quantify the strength of phase locking to the stimulus cycle as it does not consider the spread of the period histogram. For example, two period histograms, one where all spikes occur at the peak of the stimulus cycle (strong phase locking), and the other where all spikes are uniformly distributed across one stimulus half-cycle (weak phase locking), will yield the same coefficient of synchronization of 1.0.

A more sensitive measure of phase locking derived from the period histogram is the vector strength (VS (Greenwood and Durand, 1955; Goldberg and Brown, 1969)), which is identical to the synchronization index metric described by Johnson (Johnson, 1980). VS has been used extensively to quantify phase-locking strength in spike-train recordings in response to periodic stimuli (Palmer and Russell, 1986; Joris et al., 2004), including stationary speech (Young and Sachs, 1979). In this framework, each spike is treated as a complex vector that has a magnitude of 1 and an angle that is defined by the spike phase relative to the stimulus phase; VS is defined as the magnitude of the average of all such vectors for spikes pooled across all stimulus repetitions (S1 Appendix). VS is a biased estimator of the "true" vector strength (Mardia, 1972) and can reach spuriously high values at low spike counts (Yin et al., 2010). This problem is avoided by using a modification of the vector strength, called the phase-projected vector strength (VS_{pp}) (Yin et al., 2010). Similar approaches have been used in electrophysiological studies (Vinck et al., 2011). VS_{pp} differs from VS in that trial-to-trial phase consistency is also considered in computing VS_{pp} (S1 Appendix).

Overall, the period histogram and metrics derived from it (VS and VS_{pp}) work well for applications involving stationary signals with periodic TFS (e.g., tones, Fig. 1D), ENV (e.g., sinusoidally amplitude-modulated noise), or both (e.g., sinusoidally amplitude-modulated tones, Fig. 1F). However, the period histogram ignores nonstationary features in the response that arise from the auditory system. For example, spikes in the first few stimulus cycles are often ignored while constructing the period histogram to avoid the nonstationary onset response. Similarly, since spikes corresponding to different stimulus cycles are wrapped onto a single cycle, effects of adaptation are not captured in the period histogram. Moreover, its application to nonstationary or aperiodic stimuli (e.g., natural speech) is not straightforward.

Peristimulus-time-histogram (PSTH) based metrics

The single-polarity PSTH, $p(t)$, is constructed as the histogram of spike times pooled across all stimulus repetitions at a certain bin width (e.g., Fig 1C). As the PSTH shows the rate variation along the course of the stimulus, it captures the onset as well as adaption effects in the response (Kiang et al., 1965; Westerman and Smith, 1988). $p(t)$ has been applied to analyze spike trains recorded in response to periodic signals, both in the temporal and spectral domains (Young and Sachs, 1979; Delgutte, 1980; Palmer et al., 1986). A limitation of the *p(t)*-spectrum is that it is corrupted by harmonic distortions due to the rectified nature of the PSTH response (Young and Sachs, 1979). For example, the spectrum of a PSTH constructed using spike trains recorded from an AN fiber in response to a tone (F_c) can show energy at F_c as well as $2F_c$ even though the stimulus itself does not have energy at $2F_c$. These issues related to rectifier distortion can be minimized by using both polarities of the stimulus (S1 Fig). Similar to the period histogram, the PSTH can also be used to derive phase-locking metrics, such as VS and VS_{pp}. These synchrony-based metrics have been recently overshadowed by correlogram-based metrics, which are described next, since the synchrony-based metrics are limited to periodic signals. In contrast, correlogram-based approaches offer more general metrics to evaluate temporal coding of both periodic and aperiodic stimuli in the ENV/TFS dichotomy.

Interspike-interval (ISI) based approaches (e.g., correlograms)

Interspike interval histogram analyses were developed to quantify the correlation between two spike trains, either from the same neuron or from different neurons (Hagiwara, 1954; Rodieck et al., 1962; Perkel et al., 1967a,b). Interspike intervals between adjacent spikes (also called first-order intervals) within a stimulus trial are used to construct per-trial estimates of the ISI histogram, which are then averaged across trials to form the final first-order ISI histogram (Fig ST1-C). An alternative to the first-order ISI histogram, called the all-order ISI histogram (or the autocorrelogram), can be estimated in a similar way with the only difference being the inclusion of intervals between all spikes within a trial (not only adjacent spikes) to construct the histogram (Fig ST1-E) (Rodieck, 1967; Møller, 1970). The autocorrelogram has been used to study the temporal representation of stationary as well as nonstationary stimuli (Bourk, 1976; Sinex and Geisler, 1981; Cariani and Delgutte, 1996a,b). While the autocorrelogram is attractive for its simplicity, it is confounded by refractory effects (Fig ST1-E and ST1-F). In particular, since successive spikes within a single trial cannot occur within the refractory period, the autocorrelogram shows an artifactual absence of intervals for delays less than the 0.6-ms refractory period (Fig ST1-E). As a result, the autocorrelogram spectrum is partly corrupted.

Joris and colleagues extended these ISI-based analyses to remove the confounds of the refractory effects by including all-order interspike intervals *across* stimulus trials to compute a shuffled correlogram (Louage et al., 2004). A shuffled correlogram computed using spike trains in response to multiple repetitions of a single stimulus from a single neuron is called the shuffled autocorrelogram (or the SAC, Fig ST1-G). Similarly, a shuffled correlogram computed using spike trains from different neurons, or for different stimuli, is called the shuffled cross-correlogram (or the SCC). The use of across-trial all-order ISIs provides substantially more smoothing than simple all-order ISIs because many more intervals are included in the histogram (compare Fig ST1-E with Fig ST1-G, and Fig ST1-F with Fig ST1-H).

In addition, both polarities of the stimulus can be used to separate out ENV and TFS components from the response. Stimuli with alternating polarities share the same envelope, but their phases (TFS) differ by a half-cycle at all frequencies. By averaging

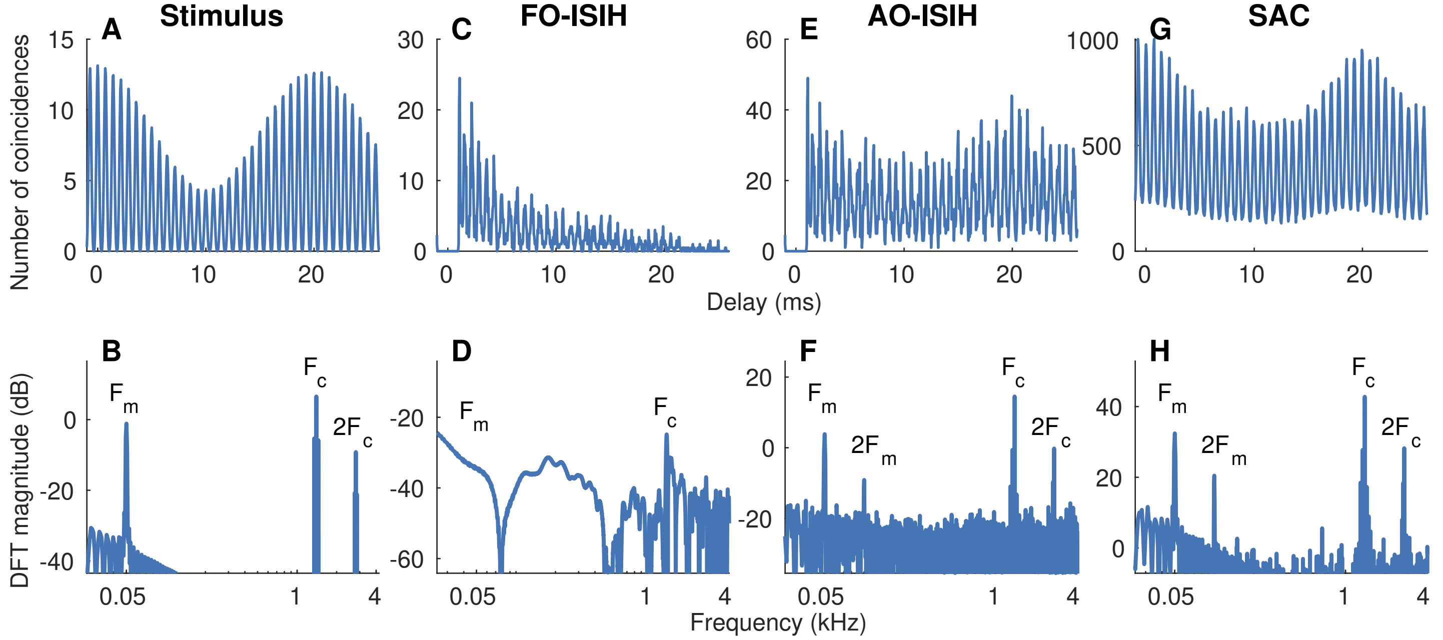

Fig ST1. The shuffled autocorrelogram is better than the first-order and all-order ISI histogram, both in the time and frequency domains. Example correlograms (top) and associated spectra (bottom) constructed using spike trains recorded from an AN fiber (CF = 1.4 kHz, medium SR) in response to a SAM-tone at F_c = CF (50-Hz modulation frequency or F_m, 0-dB modulation depth, 700-ms duration, 27 repetitions, 50 dB SPL). (A) Autocorrelation function of the half-wave rectified stimulus. (B) The discrete Fourier transform (DFT) of A. (C) The first-order (FO) ISI histogram. (D) DFT of C. The first-order ISI histogram poorly captures the carrier (TFS) and fails to capture the modulator (ENV). (E) The all-order (AO) ISI histogram. (F) DFT of E. The all-order ISI histogram captures both the carrier and modulator despite being noisy. Both the first-order (C) and the all-order (E) ISI histograms show dips for intervals less than the refractory period (∼0.6 ms), with the corresponding spectra corrupted by these refractory effects. (G) The shuffled autocorrelogram. (H) DFT of G. The shuffled autocorrelogram is smoother compared to the other correlograms, which also leads to improved SNR in the spectrum at both the carrier and modulator frequencies. All these ISI histograms are corrupted by rectifier distortion at twice the carrier frequency ($2F_c$). Bin width = 50 μs for histograms in C, E, and G.

shuffled autocorrelograms for both stimulus polarities and shuffled cross-correlograms for opposite stimulus polarities, the *polarity-tolerant* (ENV) correlogram (called the *sumcor*) is obtained (Louage et al., 2004) (S4 Appendix). Similarly, the *polarity-sensitive* (TFS) correlogram, the *difcor*, is estimated as the difference between the average autocorrelogram for both stimulus polarities and the cross-correlogram for opposite stimulus polarities (S4 Appendix). These functions have been preferred over PSTH-based analyses for estimating correlation sequences and response spectra (Joris et al., 2006; Cedolin and Delgutte, 2005; Rallapalli and Heinz, 2016). Shuffled autocorrelograms have also been used to derive temporal metrics, such as the *correlogram peak-height* and *half-width*, to quantify the strength and precision of temporal coding in the response, respectively(Louage et al., 2004), including for nonstationary stimuli (Sayles and Winter, 2008; Sayles et al., 2015; Paraouty et al., 2018). In addition, cross-correlograms have been used to develop metrics to quantify ENV/TFS similarity between responses to different stimuli recorded from the same neuron (e.g., speech stimuli (Heinz and Swaminathan, 2009; Swaminathan and Heinz, 2012; Rallapalli and Heinz, 2016)), or between responses from different neurons (Joris et al., 2006; Heinz et al., 2010; Swaminathan and Heinz, 2011).

Although correlogram-based analyses provide a rich set of temporal metrics, they

suffer from three major limitations. First, correlograms discard phase information in the response. Response phase can convey important information, especially for complex stimuli, like speech (Delgutte et al., 1998; Greenberg and Arai, 2001; Paliwal and Alsteris, 2003). Second, metrics derived from the shuffled autocorrelogram and the *sumcor* are corrupted by rectifier distortions (e.g., Fig ST1-H). Third, spectral estimates based on correlograms are appropriate for second-order stationary signals. To accommodate for nonstationary signals, usually a sliding-window-based approach is employed where in each temporal window the spectrum and/or correlogram is computed (Sayles and Winter, 2008). This windowing-based approach faces the classic problem of a time-frequency resolution trade-off. In addition, the smoothing benefit provided by the correlogram comes at large computational cost as its computation requires all-order spike-time differences across all trials. This computation cost scales quadratically (N^2) with the number of spikes (N) and can be cumbersome for large N.

References

Bourk, T. R. (1976). *Electrical responses of neural units in the anteroventral cochlear nucleus of the cat.* PhD Thesis, Massachusetts Institute of Technology.

Cariani, P. A. and Delgutte, B. (1996a). Neural correlates of the pitch of complex tones. I. Pitch and pitch salience. *Journal of Neurophysiology*, 76(3):1698–1716.

Cariani, P. A. and Delgutte, B. (1996b). Neural correlates of the pitch of complex tones. II. Pitch shift, pitch ambiguity, phase invariance, pitch circularity, rate pitch, and the dominance region for pitch. *Journal of Neurophysiology*, 76(3):1717–1734.

Cedolin, L. and Delgutte, B. (2005). Pitch of Complex Tones: Rate-Place and Interspike Interval Representations in the Auditory Nerve. *Journal of Neurophysiology*, 94(1):347–362.

Delgutte, B. (1980). Representation of speech-like sounds in the discharge patterns of auditory-nerve fibers. *The Journal of the Acoustical Society of America*, 68(3):843–857.

Delgutte, B., Hammond, B. M., and Cariani, P. A. (1998). Neural coding of the temporal envelope of speech: relation to modulation transfer functions. *Psychophysical and physiological advances in hearing*, pages 595–603.

Galambos, R. and Davis, H. (1943). The response of single auditory-nerve fibers to acoustic stimulation. *Journal of Neurophysiology*, 6(1):39–57.

Goldberg, J. M. and Brown, P. B. (1969). Response of binaural neurons of dog superior olivary complex to dichotic tonal stimuli: some physiological mechanisms of sound localization. *Journal of Neurophysiology*, 32(4):613–636.

Greenberg, S. and Arai, T. (2001). The relation between speech intelligibility and the complex modulation spectrum. In *Seventh European Conference on Speech Communication and Technology.*

Greenwood, J. A. and Durand, D. (1955). The Distribution of Length and Components of the Sum of n Random Unit Vectors. *Ann. Math. Statist.*, 26(2):233–246.

Hagiwara, S. (1954). Analysis of interval fluctuation of the sensory nerve impulse. *The Japanese Journal of Physiology*, 4:234–240.

Heinz, M. G. and Swaminathan, J. (2009). Quantifying Envelope and Fine-Structure Coding in Auditory Nerve Responses to Chimaeric Speech. *Journal of the Association for Research in Otolaryngology*, 10(3):407–423.

Heinz, M. G., Swaminathan, J., Boley, J. D., and Kale, S. (2010). Across-Fiber Coding of Temporal Fine-Structure: Effects of Noise-Induced Hearing Loss on Auditory-Nerve Responses. In Lopez-Poveda, E. A., Palmer, A. R., and Meddis, R., editors, *The Neurophysiological Bases of Auditory Perception*, pages 621–630. Springer New York.

Johnson, D. H. (1980). The relationship between spike rate and synchrony in responses of auditory-nerve fibers to single tones. *The Journal of the Acoustical Society of America*, 68(4):1115–1122.

Joris, P. X., Louage, D. H., Cardoen, L., and van der Heijden, M. (2006). Correlation Index: A new metric to quantify temporal coding. *Hearing Research*, 216–217:19–30.

Joris, P. X., Schreiner, C. E., and Rees, A. (2004). Neural Processing of Amplitude-Modulated Sounds. *Physiological Reviews*, 84(2):541–577.

Kiang, N., Watanabe, T., Thomas, E., and Clark, L. (1965). Discharge patterns of single fibers in the cat's auditory nerve. *MIT, Cambridge, MA*, 1(1):104–105.

Louage, D. H. G., Heijden, M. v. d., and Joris, P. X. (2004). Temporal Properties of Responses to Broadband Noise in the Auditory Nerve. *Journal of Neurophysiology*, 91(5):2051–2065.

Mardia, K. V. (1972). A Multi-Sample Uniform Scores Test on a Circle and its Parametric Competitor. *Journal of the Royal Statistical Society: Series B (Methodological)*, 34(1):102–113.

Møller, A. R. (1970). The Use of Correlation Analysis in Processing Neuroelectric Data. In *Progress in Brain Research*, volume 33, pages 87–99.

Paliwal, K. K. and Alsteris, L. (2003). Usefulness of Phase Spectrum in Human Speech Perception. *Eighth European Conference on Speech Communication and Technology*, page 4.

Palmer, A. R. and Russell, I. J. (1986). Phase-locking in the cochlear nerve of the guinea-pig and its relation to the receptor potential of inner hair-cells. *Hearing Research*, 24(1):1–15.

Palmer, A. R., Winter, I. M., and Darwin, C. J. (1986). The representation of steady-state vowel sounds in the temporal discharge patterns of the guinea pig cochlear nerve and primarylike cochlear nucleus neurons. *The Journal of the Acoustical Society of America*, 79(1):100–113.

Paraouty, N., Stasiak, A., Lorenzi, C., Varnet, L., and Winter, I. M. (2018). Dual Coding of Frequency Modulation in the Ventral Cochlear Nucleus. *The Journal of Neuroscience*, 38(17):4123–4137.

Perkel, D. H., Gerstein, G. L., and Moore, G. P. (1967a). Neuronal Spike Trains and Stochastic Point Processes: I. The Single Spike Train. *Biophysical Journal*, 7(4):391–418.

Perkel, D. H., Gerstein, G. L., and Moore, G. P. (1967b). Neuronal Spike Trains and Stochastic Point Processes: II. Simultaneous Spike Trains. *Biophysical Journal*, 7(4):419–440.

Rallapalli, V. H. and Heinz, M. G. (2016). Neural Spike-Train Analyses of the Speech-Based Envelope Power Spectrum Model: Application to Predicting Individual Differences with Sensorineural Hearing Loss. Trends in Hearing. 2016;20, 1–14. doi:10.1177/2331216516667319.

Rodieck, R., Kiang, N.-S., and Gerstein, G. (1962). Some Quantitative Methods for the Study of Spontaneous Activity of Single Neurons. *Biophysical Journal*, 2(4):351–368.

Rodieck, R. W. (1967). Maintained activity of cat retinal ganglion cells. *Journal of Neurophysiology*, 30(5):1043–1071.

Rose, J. E., Brugge, J. F., Anderson, D. J., and Hind, J. E. (1967). Phase-locked response to low-frequency tones in single auditory nerve fibers of the squirrel monkey. *Journal of Neurophysiology*, 30(4):769–793.

Sayles, M., Stasiak, A., and Winter, I. M. (2015). Reverberation impairs brainstem temporal representations of voiced vowel sounds: challenging "periodicity-tagged" segregation of competing speech in rooms. *Frontiers in systems neuroscience*, 8:248.

Sayles, M. and Winter, I. M. (2008). Reverberation Challenges the Temporal Representation of the Pitch of Complex Sounds. *Neuron*, 58(5):789–801.

Sinex, D. G. and Geisler, C. D. (1981). Auditory-nerve fiber responses to frequency-modulated tones. *Hearing Research*, 4(2):127–148.

Swaminathan, J. and Heinz, M. G. (2011). Predicted effects of sensorineural hearing loss on across-fiber envelope coding in the auditory nerve. *The Journal of the Acoustical Society of America*, 129(6):4001–4013.

Swaminathan, J. and Heinz, M. G. (2012). Psychophysiological Analyses Demonstrate the Importance of Neural Envelope Coding for Speech Perception in Noise. *Journal of Neuroscience*, 32(5):1747–1756.

Vinck, M., Oostenveld, R., van Wingerden, M., Battaglia, F., and Pennartz, C. M. A. (2011). An improved index of phase-synchronization for electrophysiological data in the presence of volume-conduction, noise and sample-size bias. *NeuroImage*, 55(4):1548–1565.

Westerman, L. A. and Smith, R. L. (1988). A diffusion model of the transient response of the cochlear inner hair cell synapse. *The Journal of the Acoustical Society of America*, 83(6):2266–2276.

Yin, P., Johnson, J. S., O'Connor, K. N., and Sutter, M. L. (2010). Coding of Amplitude Modulation in Primary Auditory Cortex. *Journal of Neurophysiology*, 105(2):582–600.

Young, E. D. and Sachs, M. B. (1979). Representation of steady-state vowels in the temporal aspects of the discharge patterns of populations of auditory-nerve fibers. *The Journal of the Acoustical Society of America*, 66(5):1381–1403.
