# Supplementary material for: Spectrally specific temporal analyses of spike-train responses to complex sounds: A unifying framework
Source: PLoS Comput Biol. 2021 Feb 22;17(2):e1008155. doi: 10.1371/journal.pcbi.1008155 (PMC7932515; doi:10.1371/journal.pcbi.1008155)
Supplement: S1 Appendix — (PDF) [file pcbi.1008155.s004.pdf]

S1 Appendix. Vector strength metric definitions

Vector Strength. The *vector strength* (VS) metric is used to quantify how well spikes in a spike train are synchronized to a frequency, f (Goldberg and Brown, 1969; Johnson, 1980). Let us denote a spike train with N spikes as ζ such that $\zeta = \{t_1, t_2, ..., t_N\}$ and the $\{t_i\}$s are individual spike times. To compute the vector strength, these spike times are first transformed onto the unit circle such that t_i maps to z_i as

$$z_i = e^{\jmath 2\pi f t_i}.$$

The mean of the set of complex vectors corresponding to all N spikes is

$$\rho(f) = \frac{1}{N}\sum_{i=1}^{N} z_i = \frac{1}{N}\sum_{i=1}^{N} e^{\jmath 2\pi f t_i}. \tag{A1}$$

Then, VS at frequency f is defined as the magnitude of $\rho(f)$.

$$\begin{aligned} VS(f) &= |\rho(f)| \\ &= \left|\frac{1}{N}\sum_{i=1}^{N} z_i\right| \\ &= \left|\frac{1}{N}\sum_{i=1}^{N} \left[cos(2\pi f t_i) + \jmath\ sin(2\pi f t_i)\right]\right| \\ &= \left\{\left[\frac{1}{N}\sum_{i=1}^{N} cos(2\pi f t_i)\right]^2 + \left[\frac{1}{N}\sum_{i=1}^{N} sin(2\pi f t_i)\right]^2\right\}^{\frac{1}{2}} \end{aligned} \tag{A2}$$

Phase-projected Vector Strength. The *phase-projected vector strength* (VS_{pp}) is identical to the VS for a single spike train (i.e., for a single stimulus repetition), but these metrics differ when multiple (R) stimulus repetitions are used. VS_{pp} is advantageous relative to VS when there are relatively fewer spikes per repetition (Yin et al., 2010). To estimate VS_{pp} at frequency f, the magnitude (i.e., VS) and phase $[\phi_r(f)]$ of the mean complex vector are first calculated for individual repetitions using Eqs A1 and A2 (instead of pooling spike times across all R repetitions). The per-repetition VS estimates, called $VS^r(f)$, are weighted by the cosine of the phase difference between $\phi^r(f)$ of the repetition and the mean phase based on all spikes from all repetitions, $\phi^{ref}(f)$, to estimate the *phase-projected vector strength*, $VS^r_{pp}(f)$, for the repetition.

$$VS^r_{pp}(f) = VS^r(f)\ cos\left[\phi^r(f) - \phi^{ref}(f)\right],$$

where $\phi^r(f)$ for repetition r with N_r spikes $\{t^r_1, t^r_2, ..., t^r_{N_r}\}$ is computed as

$$\phi^r(f) = \tan^{-1}\frac{\sum_{i=1}^{N_r} sin(2\pi f t^r_i)}{\sum_{i=1}^{N_r} cos(2\pi f t^r_i)},$$

and $\phi^{ref}(f)$ is computed using all spikes across all R repetitions as

$$\phi^{ref}(f) = \tan^{-1}\frac{\sum_{r=1}^{R}\sum_{i=1}^{N_r} sin(2\pi f t^r_i)}{\sum_{r=1}^{R}\sum_{i=1}^{N_r} cos(2\pi f t^r_i)}.$$

$VS_{pp}(f)$ for R repetitions is computed as the mean $VS^r_{pp}(f)$ across all repetitions,

$$VS_{pp}(f) = \frac{1}{R}\sum_{i=1}^{R} VS^r_{pp}(f).$$

References

Goldberg, J. M. and Brown, P. B. (1969). Response of binaural neurons of dog superior olivary complex to dichotic tonal stimuli: some physiological mechanisms of sound localization. *Journal of Neurophysiology*, 32(4):613–636.

Johnson, D. H. (1980). The relationship between spike rate and synchrony in responses of auditory-nerve fibers to single tones. *The Journal of the Acoustical Society of America*, 68(4):1115–1122.

Yin, P., Johnson, J. S., O'Connor, K. N., and Sutter, M. L. (2010). Coding of Amplitude Modulation in Primary Auditory Cortex. *Journal of Neurophysiology*, 105(2):582–600.
