# Supplementary material for: Spectrally specific temporal analyses of spike-train responses to complex sounds: A unifying framework
Source: PLoS Comput Biol. 2021 Feb 22;17(2):e1008155. doi: 10.1371/journal.pcbi.1008155 (PMC7932515; doi:10.1371/journal.pcbi.1008155)
Supplement: S2 Appendix — (PDF) [file pcbi.1008155.s005.pdf]

S2 Appendix. Relation between the *vector strength* metric and the *difference PSTH*

Let us assume that we have R sets of spike trains $\{\underline{\zeta}_i\} : i \in [1, .., R]$ for a tone stimulus with duration D and frequency f_0. Let the corresponding PSTH be $p(t)$, and the total number of spikes be N.

In Eq A1, $\sum_{i=1}^{N} e^{\jmath 2\pi f t_i}$ can be written as (van Hemmen, 2013)

$$\sum_{i=1}^{N} e^{\jmath 2\pi f t_i} = \int_{t=0}^{D} p(t) e^{\jmath 2\pi f t} dt. \tag{A3}$$

Using Eq A3 in Eq A1, we get

$$\begin{aligned} \rho(f) &= \frac{1}{N} \int_{t=0}^{D} p(t) e^{\jmath 2\pi f t} dt \\ \implies \rho(f_0) &= \frac{1}{N} \int_{t=0}^{D} p(t) e^{\jmath 2\pi f_0 t} dt. \end{aligned} \tag{A4}$$

If we assume response phase locking to positive and negative polarity of a sinusoid (f_0) differ by a phase of π [i.e., a time difference of $T_0/2 (= 1/2f_0)$ such that $p(t) \simeq n(t) e^{\jmath 2\pi f T_0/2}$], we can write

$$\begin{aligned} \rho(f) &= \frac{1}{N} \int_{t=0}^{D} p(t) e^{\jmath 2\pi f t} dt \\ &= \frac{1}{N} \int_{t=0}^{D} n(t) e^{\jmath 2\pi f T_0/2} e^{\jmath 2\pi f t} dt. \end{aligned} \tag{A5}$$

For $f \neq f_0$, the integral in Eq A5 will be zero. For $f = f_0$,

$$\begin{aligned} \rho(f_0) &= \frac{1}{N} \int_{t=0}^{D} n(t) e^{\jmath 2\pi f_0 \frac{1}{f_0} \frac{1}{2}} e^{\jmath 2\pi f_0 t} dt \\ &= \frac{1}{N} \int_{t=0}^{D} n(t) e^{\jmath \pi} e^{\jmath 2\pi f_0 t} dt \\ \implies \rho(f_0) &= \frac{1}{N} \int_{t=0}^{D} -n(t) e^{\jmath 2\pi f_0 t} dt. \end{aligned} \tag{A6}$$

Adding Eqs. A4 and A6, we get

$$\begin{aligned} 2\rho(f_0) &= \frac{1}{N} \int_{t=0}^{D} [p(t) - n(t)] e^{\jmath 2\pi f_0 t} dt \\ &= \frac{1}{N} \int_{t=0}^{D} 2d(t) e^{\jmath 2\pi f_0 t} dt \\ \implies \rho(f_0) &= \frac{1}{N} \int_{t=0}^{D} d(t) e^{\jmath 2\pi f_0 t} dt \\ &= \frac{D(-f_0)}{N}, \end{aligned}$$

where $D(f) = \int_{t=0}^{D} d(t) e^{-\jmath 2\pi f t} dt$ is the Fourier transform of $d(t)$. Since $d(t)$ is a real signal, $|D(f)| = |D(-f)|$. Thus, the relation between VS and the difference PSTH becomes,

$$VS(f) = |\rho(f)| = \frac{|D(f)|}{N}. \tag{A7}$$

References

van Hemmen, J. L. (2013). Vector strength after Goldberg, Brown, and von Mises: biological and mathematical perspectives. *Biological cybernetics*, 107(4):385–396.
