# Supplementary material for: Spectrally specific temporal analyses of spike-train responses to complex sounds: A unifying framework
Source: PLoS Comput Biol. 2021 Feb 22;17(2):e1008155. doi: 10.1371/journal.pcbi.1008155 (PMC7932515; doi:10.1371/journal.pcbi.1008155)
Supplement: S3 Appendix — (PDF) [file pcbi.1008155.s006.pdf]

S3 Appendix. Relation between *shuffled correlograms* and *apPSTHs*

Consider $\mathbb{X}$: a set of T_X spike trains $\{\underline{\zeta_1}, \underline{\zeta_2}, ..., \underline{\zeta_{T_X}}\}$ in response to a stimulus of duration D. For each spike train $\underline{\zeta_i}$, we can construct a PSTH, $\underline{x_i}$, with PSTH bin width Δ so that the length of the single-trial PSTH $\underline{x_i}$ is $M = D/\Delta$. The single-trial PSTH is a binary-valued vector because each element in the vector is either 0 or 1. Let us denote the PSTH for $\mathbb{X}$ by $PSTH_X$ such that $PSTH_X = \sum_{i=1}^{T_X} \underline{x_i}$. Consider $\mathbb{Y}$: another set of T_Y spike trains, with $\underline{y_i}$ and $PSTH_Y$ defined similarly to $\underline{x_i}$ and $PSTH_X$, respectively. Let us assume that the stimulus duration and bin width for $\underline{y_i}$ are the same as that for $\underline{x_i}$. Let the average discharge rates (in spikes/s) for $\mathbb{X}$ and $\mathbb{Y}$ be r_X and r_Y, respectively. The shuffled cross-correlogram (SCC) for two spike trains $\underline{\zeta_i}$ and $\underline{\zeta_j}$ computed using tallying (Louage et al., 2004) is identical to the cross-correlation function (denoted by $\mathcal{R}_{\mathcal{XY}}$) between their respective PSTHs, ($\underline{x_i}$ and $\underline{x_j}$). Thus, the raw (not normalized) shuffled cross-correlogram (SCC^{raw}) at τ delay can be computed as

$$\begin{aligned}
SCC^{raw}_{\mathbb{X},\mathbb{Y}}(\tau) &= \mathcal{R}_{\mathcal{XY}}(\underline{x_1}, \{\underline{y_1}, \underline{y_2}, ..., \underline{y_{T_Y}}\}) + ... + \mathcal{R}_{\mathcal{XY}}(\underline{x_{T_X}}, \{\underline{y_1}, \underline{y_2}, ..., \underline{y_{T_Y}}\}) \\
&= \mathcal{R}_{\mathcal{XY}}(\underline{x_1}, [\underline{y_1} + \underline{y_2} + ... + \underline{y_{T_Y}}]) + ...+ \qquad \text{(A8)} \\
&\quad \mathcal{R}_{\mathcal{XY}}(\underline{x_{T_X}}, [\underline{y_1} + \underline{y_2} + ... + \underline{y_{T_Y}}]) \\
&= \sum_{i=1}^{T_X} \sum_{j=1}^{T_Y} \mathcal{R}_{\mathcal{XY}}(\underline{x_i}, \underline{y_j}) \\
&= \mathcal{R}_{\mathcal{XY}}(PSTH_X, PSTH_Y) \\
\implies SCC^{norm}_{\mathbb{X},\mathbb{Y}}(\tau) &= \frac{\mathcal{R}_{\mathcal{XY}}(PSTH_X, PSTH_Y)}{T_X T_Y r_X r_Y D\Delta}, \qquad \text{(A9)}
\end{aligned}$$

where SCC^{norm} is the normalized SCC (Louage et al., 2004; Heinz and Swaminathan, 2009).

Similarly, the raw shuffled autocorrelogram (SAC^{raw}) at τ delay can be computed as,

$$\begin{aligned}
SAC^{raw}_{\mathbb{X}}(\tau) &= \mathcal{R}_{\mathcal{XY}}(\underline{x_1}, \{\underline{x_2}, \underline{x_3}, ..., \underline{x_{T_X}}\}) + \mathcal{R}_{\mathcal{XY}}(\underline{x_2}, \{\underline{x_1}, \underline{x_3}, ..., \underline{x_{T_X}}\}) + ... \\
&\quad + \mathcal{R}_{\mathcal{XY}}(\underline{x_{T_X}}, \{\underline{x_1}, \underline{x_2}, ... + \underline{x_{T_X-1}}\}) \\
&= \mathcal{R}_{\mathcal{XY}}(\underline{x_1}, [\underline{x_2} + \underline{x_3} + ... + \underline{x_{T_X}}]) + \mathcal{R}_{\mathcal{XY}}(\underline{x_2}, [\underline{x_1} + \underline{x_3} + ... + \underline{x_{T_X}}]) \\
&\quad + ... + \mathcal{R}_{\mathcal{XY}}(\underline{x_{T_X}}, [\underline{x_1} + \underline{x_2} + ... + \underline{x_{T_X-1}}]) \\
&= \sum_{i=1}^{T_X} \sum_{j=1, j\neq i}^{T_X} \mathcal{R}_{\mathcal{XY}}(\underline{x_i}, \underline{x_j}) \\
&= \sum_{i=1}^{T_X} \sum_{j=1}^{T_X} \mathcal{R}_{\mathcal{XY}}(\underline{x_i}, \underline{x_j}) - \sum_{i=1}^{T_X} \mathcal{R}_{\mathcal{XY}}(\underline{x_i}, \underline{x_i}) \\
&= \mathcal{R}_{\mathcal{X}}(PSTH_X) - \sum_{i=1}^{T_X} \mathcal{R}_{\mathcal{X}}(\underline{x_i}) \\
\implies SAC^{norm}_{\mathbb{X}}(\tau) &= \frac{\mathcal{R}_{\mathcal{X}}(PSTH_X) - \sum_{i=1}^{T_X} \mathcal{R}_{\mathcal{X}}(\underline{x_i})}{T_X(T_X - 1) r_X^2 D\Delta}, \qquad \text{(A10)}
\end{aligned}$$

where $\mathcal{R}_{\mathcal{X}}$ denotes the autocorrelation function. Similar to autocorrelation functions, the SAC^{norm} has its maximum at zero delay.

In the numerator of Eq A10, the term $\sum_{i=1}^{T_X} \mathcal{R}_{\mathcal{X}}(\underline{x_i})$ is negligible compared to $\mathcal{R}_{\mathcal{X}}(PSTH_X)$ for $\tau \neq 0$. For $\tau = 0$, $\sum_{i=1}^{T_X} \mathcal{R}_{\mathcal{X}}(\underline{x_i})$ is equal to the total number of spikes

(N) in $\mathbb{X}$. Thus, Eq A10 can be further approximated by,

$$SAC_{\mathbb{X}}^{norm}(\tau) \simeq \frac{\mathcal{R}_{\mathcal{X}}(PSTH_X) - N\delta(\tau)}{T_X(T_X-1)r_X^2 D\Delta} \tag{A11}$$

$$= \frac{\mathcal{R}_{\mathcal{X}}(PSTH_X)}{T_X(T_X-1)r_X^2 D\Delta} - \frac{\delta(\tau)}{(T_X-1)r_X\Delta}$$

$$\simeq \frac{\mathcal{R}_{\mathcal{X}}(PSTH_X)}{T_X^2 r_X^2 D\Delta} - \frac{\delta(\tau)}{T_X r_X \Delta} \tag{A12}$$

where $N = r_X D T_X$, and δ is the Dirac delta function. The simplifying approximation in Eq A12 is valid for typically used T_X values in neurophysiological experiments, and equates the normalization factors between $SACs$ and $SCCs$ when working with *difcor* and *sumcor* (e.g., S4 Appendix). Eqs A9 to A12 indicate that correlograms can be computed much more efficiently using *apPSTHs* instead of by tallying spike times [$\mathcal{O}(N)$ instead of $\mathcal{O}(N^2)$, see main text].

References

Louage, D. H. G., Heijden, M. v. d., and Joris, P. X. (2004). Temporal Properties of Responses to Broadband Noise in the Auditory Nerve. *Journal of Neurophysiology*, 91(5):2051–2065.

Heinz, M. G. and Swaminathan, J. (2009). Quantifying Envelope and Fine-Structure Coding in Auditory Nerve Responses to Chimaeric Speech. *Journal of the Association for Research in Otolaryngology*, 10(3):407–423.
