# Supplementary material for: Spectrally specific temporal analyses of spike-train responses to complex sounds: A unifying framework
Source: PLoS Comput Biol. 2021 Feb 22;17(2):e1008155. doi: 10.1371/journal.pcbi.1008155 (PMC7932515; doi:10.1371/journal.pcbi.1008155)
Supplement: S4 Appendix — (PDF) [file pcbi.1008155.s007.pdf]

S4 Appendix. Relation between *difcor/sumcor* and *difference/sum PSTHs*

Consider $\mathbb{X}_+$: spike trains in response to the positive polarity of a stimulus, and $\mathbb{X}_-$: spike trains in response to the negative polarity of the stimulus. Then, the *difcor* at τ delay can be computed as

$$\begin{aligned} difcor_{\mathbb{X}}(\tau) &= \frac{1}{2}\left[\frac{SAC^{norm}_{\mathbb{X}_+} + SAC^{norm}_{\mathbb{X}_-}}{2} - \frac{SCC^{norm}_{\mathbb{X}_+,\mathbb{X}_-} + SCC^{norm}_{\mathbb{X}_-,\mathbb{X}+}}{2}\right] \\ &= \frac{1}{4}\left[SAC^{norm}_{\mathbb{X}_+} + SAC^{norm}_{\mathbb{X}_-} - SCC^{norm}_{\mathbb{X}_+,\mathbb{X}_-} - SCC^{norm}_{\mathbb{X}_-,\mathbb{X}+}\right] \end{aligned}$$

For analytic simplicity, we use Eq A12 for SAC^{norm} instead of Eq A10. Let us assume that the number of repetitions and average rates for both polarities are the same. Thus,

$$\begin{aligned} difcor_{\mathbb{X}}(\tau) = \frac{1}{4\mathcal{K}}[&\mathcal{R}_{\mathcal{X}}(PSTH_{\mathbb{X}_+}) - N\delta(\tau) + \mathcal{R}_{\mathcal{X}}(PSTH_{\mathbb{X}_-}) - N\delta(\tau) \\ &- \mathcal{R}_{\mathcal{X}\mathcal{Y}}(PSTH_{\mathbb{X}_+}, PSTH_{\mathbb{X}_-}) - \mathcal{R}_{\mathcal{X}\mathcal{Y}}(PSTH_{\mathbb{X}_-}, PSTH_{\mathbb{X}_+})], \end{aligned}$$

where $\mathcal{K} = T_X^2 r_X^2 D\Delta$ is a constant. Now, $PSTH_{\mathbb{X}_+} = p(t)$, $PSTH_{\mathbb{X}_-} = n(t)$, and the difference PSTH $d(t) = [p(t) - n(t)]/2$. Then, the *difcor* for $\mathbb{X}$ at delay τ is

$$\begin{aligned} difcor_{\mathbb{X}}(\tau) = &\frac{1}{4\mathcal{K}}\left\{\mathcal{R}_{\mathcal{X}}[p(t)] + \mathcal{R}_{\mathcal{X}}[n(t)] - \mathcal{R}_{\mathcal{X}\mathcal{Y}}[p(t), n(t)] - \mathcal{R}_{\mathcal{X}\mathcal{Y}}[n(t), p(t)]\right\} \\ &- \frac{N\delta(\tau)}{2\mathcal{K}} \end{aligned}$$

Now,

$$\begin{aligned} &\mathcal{R}_{\mathcal{X}}[p(t)] + \mathcal{R}_{\mathcal{X}}[n(t)] - \mathcal{R}_{\mathcal{X}\mathcal{Y}}[p(t), n(t)] - \mathcal{R}_{\mathcal{X}\mathcal{Y}}[n(t), p(t)] \\ &= \int_{t=0}^{D} p(t)p(t-\tau)dt + \int_{t=0}^{D} n(t)n(t-\tau)dt - \int_{t=0}^{D} p(t)n(t-\tau)dt - \int_{t=0}^{D} n(t)p(t-\tau)dt \\ &= \int_{t=0}^{D} p(t)[p(t-\tau) - n(t-\tau)]dt - \int_{t=0}^{D} n(t)[p(t-\tau) - n(t-\tau)]dt \\ &= \int_{t=0}^{D} 2p(t)d(t-\tau)dt - \int_{t=0}^{D} 2n(t)d(t-\tau)dt \\ &= \int_{t=0}^{D} 2[p(t) - n(t)]d(t-\tau)dt \\ &= \int_{t=0}^{D} 4d(t)d(t-\tau)dt \\ &= 4\mathcal{R}_{\mathcal{X}}[d(t)] \end{aligned}$$

Thus,

$$\begin{aligned}
difcor_{\mathbb{X}}(\tau) &= \frac{1}{4\mathcal{K}} \left\{ \mathcal{R}_{\mathcal{X}}\left[p(t)\right] + \mathcal{R}_{\mathcal{X}}\left[n(t)\right] - \mathcal{R}_{\mathcal{X}\mathcal{Y}}\left[p(t), n(t)\right] - \mathcal{R}_{\mathcal{X}\mathcal{Y}}\left[n(t), p(t)\right] \right\} \\
&\quad - \frac{N\delta(\tau)}{2\mathcal{K}} \\
&= \frac{1}{4\mathcal{K}} \times 4\mathcal{R}_{\mathcal{X}}[d(t)] - \frac{N\delta(\tau)}{2\mathcal{K}} \\
&= \frac{\mathcal{R}_{\mathcal{X}}[d(t)]}{\mathcal{K}} - \frac{N\delta(\tau)}{2\mathcal{K}} \\
&= \frac{\mathcal{R}_{\mathcal{X}}[d(t)]}{T_X^2 r_X^2 D\Delta} - \frac{r_X D T_X \delta(\tau)}{2T_X^2 r_X^2 D\Delta} \\
\implies difcor_{\mathbb{X}}(\tau) &= \frac{\mathcal{R}_{\mathcal{X}}[d(t)]}{T_X^2 r_X^2 D\Delta} - \frac{\delta(\tau)}{2T_X r_X \Delta} \qquad \text{(A13)}
\end{aligned}$$

Similarly, it can be shown that

$$\begin{aligned}
sumcor_{\mathbb{X}}(\tau) &= \frac{1}{2}\left[SAC_{\mathbb{X}}^{norm} + SCC_{\mathbb{X}_+,\mathbb{X}_-}^{norm} \right] \\
&= \frac{1}{2}\left[\frac{SAC_{\mathbb{X}_+}^{norm} + SAC_{\mathbb{X}_-}^{norm}}{2} + \frac{SCC_{\mathbb{X}_+,\mathbb{X}_-}^{norm} + SCC_{\mathbb{X}_-,\mathbb{X}_+}^{norm}}{2} \right] \\
&= \frac{1}{4\mathcal{K}} \times 4\mathcal{R}_{\mathcal{X}}[s(t)] - \frac{N\delta(\tau)}{2\mathcal{K}} \\
&= \frac{\mathcal{R}_{\mathcal{X}}[s(t)]}{\mathcal{K}} - \frac{N\delta(\tau)}{2\mathcal{K}} \\
\implies sumcor_{\mathbb{X}}(\tau) &= \frac{\mathcal{R}_{\mathcal{X}}[s(t)]}{T_X^2 r_X^2 D\Delta} - \frac{\delta(\tau)}{2T_X r_X \Delta} \qquad \text{(A14)}
\end{aligned}$$

where $s(t)$ is the sum PSTH, i.e., $s(t) = [p(t) + n(t)]/2$.

Eqs A13 and A14 indicate that *sumcor* and *difcor* are related to the autocorrelation function of the *sum* and *difference* PSTHs, respectively, and thus can be computed much more efficiently [$\mathcal{O}(N)$ rather than $\mathcal{O}(N^2)$].
