# Supplementary material for: Spectrally specific temporal analyses of spike-train responses to complex sounds: A unifying framework
Source: PLoS Comput Biol. 2021 Feb 22;17(2):e1008155. doi: 10.1371/journal.pcbi.1008155 (PMC7932515; doi:10.1371/journal.pcbi.1008155)
Supplement: S5 Appendix — (PDF) [file pcbi.1008155.s008.pdf]

S5 Appendix. Relation between *shuffled-correlogram* peak-height and *apPSTHs*

Consider a difference PSTH, $d(t)$, based on a set of spike trains $\mathbb{X}$ in response to a stimulus of duration D. Let us denote the Fourier transform of $d(t)$ by $D(f)$. Then, from Eq A13, the *difcor* peak-height, i.e., *difcor* value at zero delay (τ), can be computed as

$$
\begin{aligned}
difcor_X(\tau = 0) &= \left.\frac{\mathcal{R}_{\mathcal{X}}\{d(t)\}}{\mathcal{K}}\right|_{\tau=0} - \left.\frac{N\delta(\tau)}{2\mathcal{K}}\right|_{\tau=0} \\
&= \frac{1}{\mathcal{K}} \int_{t=0}^{D} d^2(t)dt - \frac{N}{2\mathcal{K}} \\
&= \frac{1}{\mathcal{K}} \int_{f=-\infty}^{\infty} |D(f)|^2 \, df - \frac{N}{2\mathcal{K}}, \quad \text{(by Parseval's theorem)}
\end{aligned} \tag{A15}
$$

Following similar steps from Eq A14, it can also be shown that the *sumcor* peak-height can be computed as

$$
sumcor_X(\tau = 0) = \frac{1}{\mathcal{K}} \int_{f=-\infty}^{\infty} |S(f)|^2 \, df - \frac{N}{2\mathcal{K}} \tag{A16}
$$

where $S(f)$ is the Fourier transform of the sum PSTH, $s(t)$.

Comparing Eq A7 with Eqs. A15 and A16, we see that vector strength is a frequency-specific metric, whereas correlogram peak-heights are broadband measures, which are thus susceptible to rectifier distortion (see Fig 5).
