# Supplementary material for: Spectrally specific temporal analyses of spike-train responses to complex sounds: A unifying framework
Source: PLoS Comput Biol. 2021 Feb 22;17(2):e1008155. doi: 10.1371/journal.pcbi.1008155 (PMC7932515; doi:10.1371/journal.pcbi.1008155)
Supplement: S1 Table — (PDF) [file pcbi.1008155.s009.pdf]

S1 Table. Glossary of terms and definitions

Table 1. Glossary of terms and definitions.

Term	Definition
Electrophysiology	Studies that record and analyze far-field (gross) potentials, e.g., electroencephalography
Neurophysiology	Studies that record and analyze spike-train data from neurons, e.g., AN fiber spike trains
Stationarity	A signal is stationary when the signal parameters do not change over time. For example, a stochastic signal like white Gaussian noise is stationary if the amplitude probability density function is constant across time. Similarly, a deterministic pure tone can be considered an example of a stationary sinusoidal process with a particular amplitude, frequency, and initial phase.
Second-order stationarity	A stochastic signal is second-order stationary if its mean and autocorrelation function do not change over time. Second-order stationarity is also referred to as wide-sense stationarity.
Linearity	A system is linear if it obeys the rules of superposition. For example, consider a system for which inputs x_1 and x_2 evoke responses y_1 and y_2, respectively. Then, the system is linear if the response to input $ax_1 + bx_2$ is $ay_1 + by_2$. An auditory corollary of linearity is that a linear system (e.g., the ear canal) processes sound in the same way at soft and loud sound levels, which means that for every dB increase in the input, the output is increased by the same dB.
Time invariance	A system is time invariant if its parameters (e.g., gain at all frequencies) do not change over time
Periodic signal	A perfectly repeating signal, e.g., a tone, or a synthetic vowel with constant pitch
Aperiodic signal	A signal that does not repeat, e.g., white Gaussian noise
Polarity-tolerant response	Response component that does not depend on stimulus polarity, e.g., the onset response
Polarity-sensitive response	Response component that depends on stimulus polarity, e.g., phase-locked spike trains in response to a low-frequency tone
Even sequence	$x[n]$ is even if $x[n] = x[-n]$
Odd sequence	$x[n]$ is odd if $x[n] = -x[-n]$

List of terms with definitions that are frequently used in the present report.
