# Supplementary material for: Spectrally specific temporal analyses of spike-train responses to complex sounds: A unifying framework
Source: PLoS Comput Biol. 2021 Feb 22;17(2):e1008155. doi: 10.1371/journal.pcbi.1008155 (PMC7932515; doi:10.1371/journal.pcbi.1008155)
Supplement: S2 Table — (PDF) [file pcbi.1008155.s010.pdf]

S2 Table. Parameters for the AN model

Table 2. AN model parameters.

Parameter	Value
Sampling Frequency	100 kHz
Number of Repetitions (per polarity)	25
Spontaneous firing rate (SR)	70 spikes/s
Absolute refractory period	0.6 ms
Baseline mean relative refractory period	0.6 ms
OHC health value	1.0 (normal)
IHC health value	1.0 (normal)
Species	1 (cat)
Fractional Gaussian noise type	0 (fixed)
Implementation type of the power-law functions in the Synapse	0 (approximate)
Spike time resolution	10 μs

List of parameters used in the auditory-nerve (AN) model to generate simulated spike-train data.
