# Supplementary material for: Spectrally specific temporal analyses of spike-train responses to complex sounds: A unifying framework
Source: PLoS Comput Biol. 2021 Feb 22;17(2):e1008155. doi: 10.1371/journal.pcbi.1008155 (PMC7932515; doi:10.1371/journal.pcbi.1008155)
Supplement: S1 Fig — (PDF) [file pcbi.1008155.s011.pdf]

S1 Fig. Graphical illustration of *apPSTHs* in Table 1

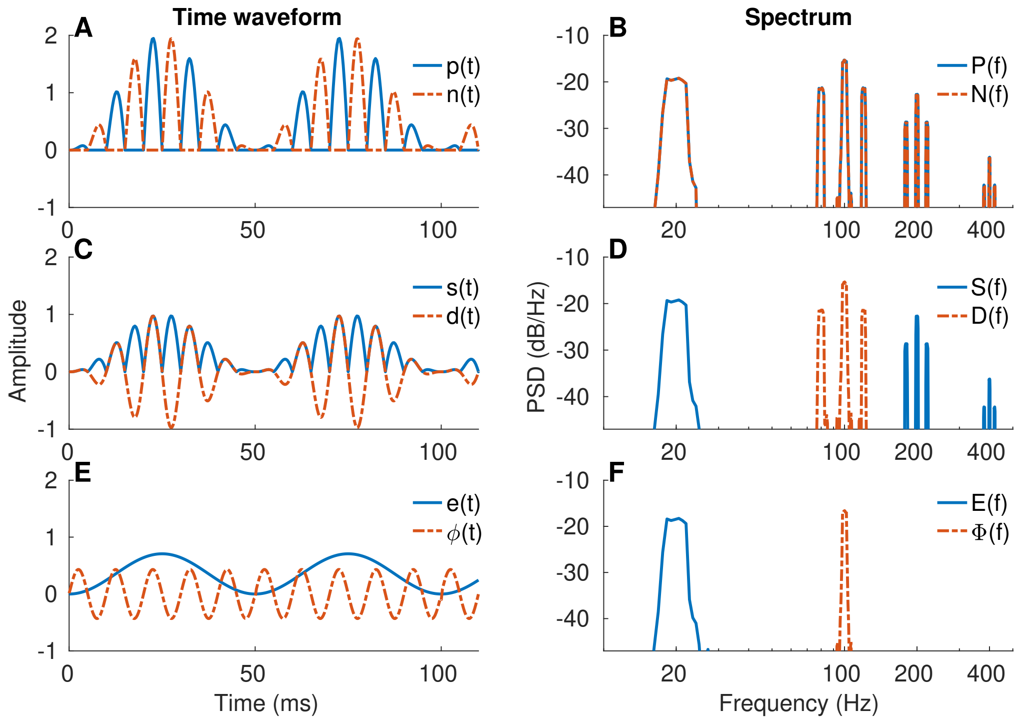

S1 Fig. Graphical illustration of *apPSTHs* in Table 1. Graphical illustration for several *apPSTHs* for a simple half-wave rectifying model. A SAM tone (carrier = 100 Hz, modulation frequency = 20 Hz, sampling frequency = 3 kHz, duration = 1 s) was used as the stimulus, although for clarity only the first 100-ms are shown in time. Note that rectifier distortions occur at even harmonics of the carrier for $P(f)$, $N(f)$, and $S(f)$, but not for $D(f)$, $E(f)$, or $\Phi(f)$.
