# Supplementary material for: Spectrally specific temporal analyses of spike-train responses to complex sounds: A unifying framework
Source: PLoS Comput Biol. 2021 Feb 22;17(2):e1008155. doi: 10.1371/journal.pcbi.1008155 (PMC7932515; doi:10.1371/journal.pcbi.1008155)
Supplement: S2 Fig — (PDF) [file pcbi.1008155.s012.pdf]

S2 Fig. Nonlinear inner-hair-cell transduction function introduces additional sidebands in the spectrum for a SAM tone.

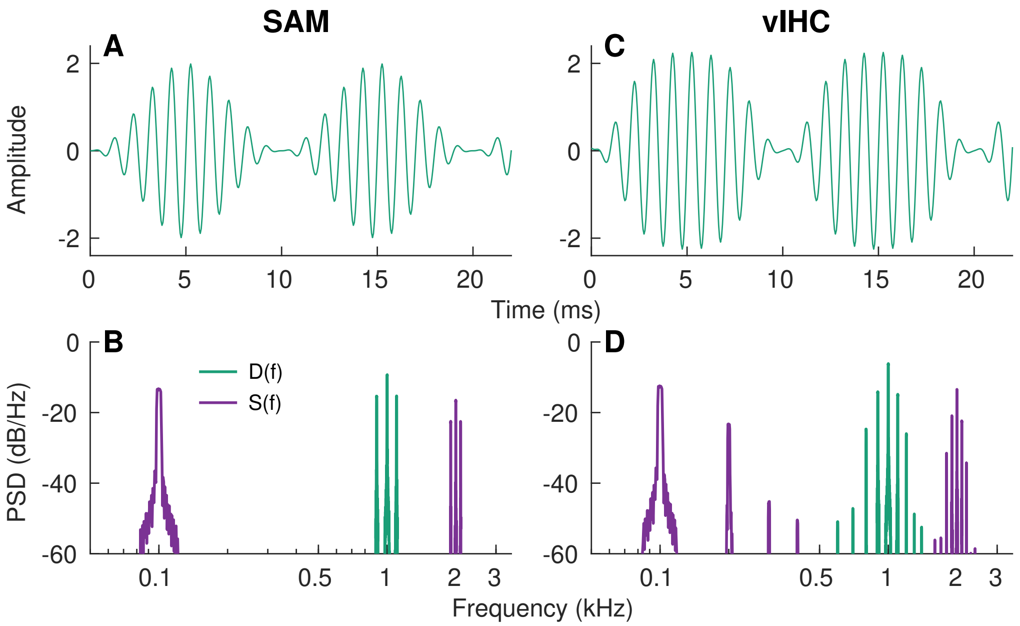

S2 Fig. Nonlinear inner-hair-cell transduction function introduces additional sidebands in the spectrum for a SAM tone. (A) Waveform for a SAM tone (F_c=1 kHz, F_m=100 Hz, 0-dB modulation depth). (B) $D(f)$ and $S(f)$ for the SAM tone in A. (C) Waveform of the output after processing the SAM tone through a sigmoid function. The sigmoid function was used as a simple proxy for the inner-hair-cell transduction function. This output (vIHC) was further low-pass filtered at 2 kHz to mimic the membrane properties of inner hair cells. (D) $D(f)$ and $S(f)$ for the signal in C. In addition to having power at F_c and $F_c \pm F_m$, $D(f)$ for vIHC has substantial energy at $F_c \pm 2F_m$ (plus reduced energy at higher multiple F_m-offsets from F_c). Similarly, $S(f)$ for vIHC has substantial energy at F_m as well as at the first few harmonics of F_m. $S(f)$ is also corrupted by rectifier distortion at $2F_c$ (and multiple F_m-offsets from $2F_c$) as expected.
