# Supplementary material for: Spectrally specific temporal analyses of spike-train responses to complex sounds: A unifying framework
Source: PLoS Comput Biol. 2021 Feb 22;17(2):e1008155. doi: 10.1371/journal.pcbi.1008155 (PMC7932515; doi:10.1371/journal.pcbi.1008155)
Supplement: S3 Fig — (PDF) [file pcbi.1008155.s013.pdf]

S3 Fig. Neural characterization of ENV and TFS using *apPSTHs* for a synthesized stationary vowel

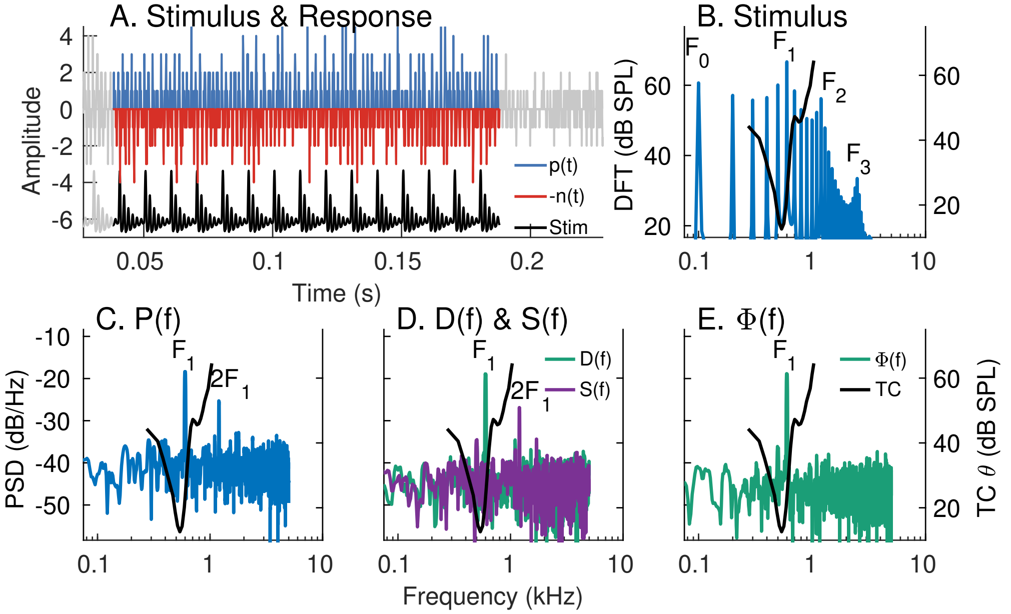

S3 Fig. Spectral-domain application of various *apPSTHs* to spike trains recorded in response to a stationary vowel. Example of spectral analyses of spike trains recorded from an AN fiber (CF= 530 Hz, SR=90 spikes/s) in response to a synthesized stationary vowel (s_1 described in *Materials and Methods*, fundamental frequency: $F_0 = 100$ Hz, first formant: $F_1 = 600$ Hz). (A) Time-domain representation of $p(t)$, $n(t)$, and the stimulus (*Stim*). $n(t)$ is reflected across the x-axis for display. Signals outside the analysis window are shown in gray. PSTH bin width = 0.1 ms. Number of stimulus repetitions per polarity = 30. Stimulus intensity = 65 dB SPL. (B) Stimulus spectrum (blue, left yaxis). In panels B-E, the frequency-threshold tuning curve (TC θ, black) of the neuron is plotted on the right y-axis. The neuron's CF was close to the first stimulus formant. (C) $P(f)$, which shows a strong response to the 6th harmonic (first formant) and the 12th harmonics (due to rectifier distortion). (D) Spectra for difference [$D(f)$, green] and sum [$S(f)$, purple] PSTHs. $D(f)$ shows a clear peak at the 6th harmonic and little energy near the 12th harmonic. Similar to $P(f)$, $S(f)$ shows substantial energy at twice the TFS (F_1) frequency due to rectifier distortion. (E) Spectra of Hilbert-based TFS PSTH [$\Phi(f)$, green]. $P(f)$ and $S(f)$ are corrupted by rectifier distortion at $2F_1$ frequency. The response primarily reflects TFS-based F_1 coding (E) and little envelope coding (D), which is consistent with the "synchrony-capture" phenomenon for stationary vowel coding (Young and Sachs, 1979; Delgutte and Kiang, 1984). Note that $E(f)$ is not shown because $e(t)$ was essentially flat across the vowel duration, and therefore had little energy other than at 0 Hz.

References

Young, E. D. and Sachs, M. B. (1979). Representation of steady-state vowels in the temporal aspects of the discharge patterns of populations of auditory-nerve fibers. *The Journal of the Acoustical Society of America*, 66(5):1381–1403.

Delgutte, B. and Kiang, N. Y. S. (1984). Speech coding in the auditory nerve: I. Vowel-like sounds. *The Journal of the Acoustical Society of America*, 75(3):866–878.
