# Supplementary material for: Spectrally specific temporal analyses of spike-train responses to complex sounds: A unifying framework
Source: PLoS Comput Biol. 2021 Feb 22;17(2):e1008155. doi: 10.1371/journal.pcbi.1008155 (PMC7932515; doi:10.1371/journal.pcbi.1008155)
Supplement: S4 Fig — (PDF) [file pcbi.1008155.s014.pdf]

S4 Fig. DFT-magnitude for the nonstationary vowel, s_2.

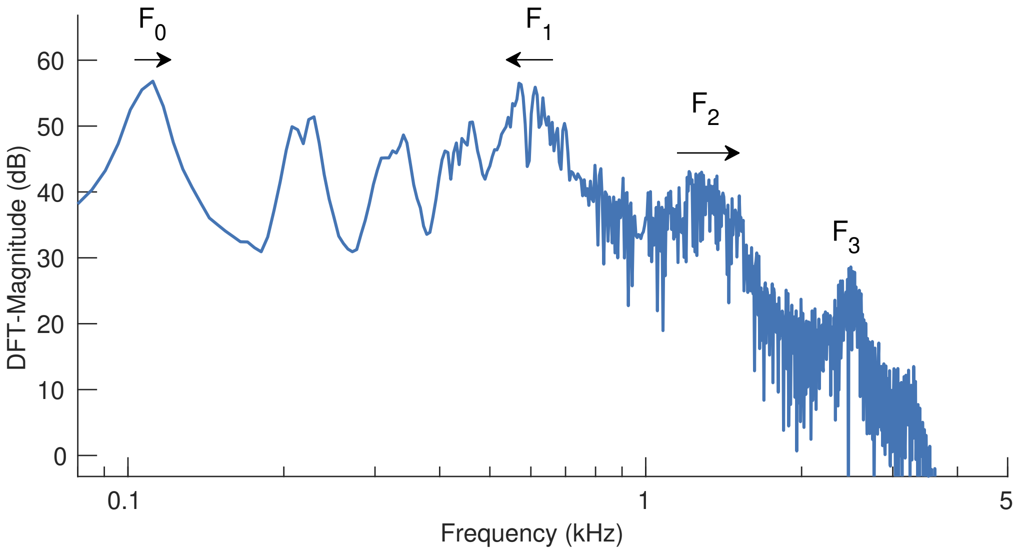

S4 Fig. DFT-magnitude for the nonstationary vowel, s_2. The stimulus duration was 188 ms. The movements of F_0 (100 to 120 Hz), F_1 (630 to 570 Hz), and F_2 (1200 to 1500 Hz) are indicated by arrows. F_3 was fixed at 2500 Hz.
