# Supplementary material for: Spectrally specific temporal analyses of spike-train responses to complex sounds: A unifying framework
Source: PLoS Comput Biol. 2021 Feb 22;17(2):e1008155. doi: 10.1371/journal.pcbi.1008155 (PMC7932515; doi:10.1371/journal.pcbi.1008155)
Supplement: S5 Fig — (PDF) [file pcbi.1008155.s015.pdf]

S5 Fig. FFR harmonicgram constructed using the Hilbert-phase FFR.

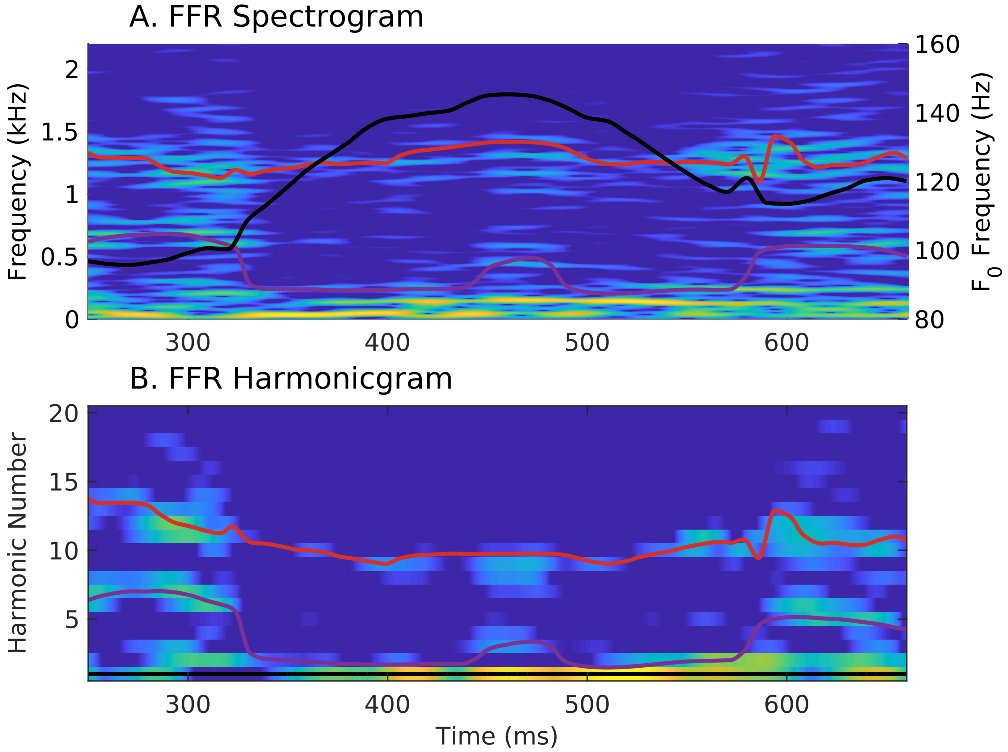

S5 Fig. FFR harmonicgram can be constructed using the Hilbert-phase response. Same format as Fig 12. The spectrogram (A) and the harmonicgram (B) were constructed using $\phi(t)$.
